# Supplementary material for: Diversity of oligomerization in Drosophila semaphorins suggests a mechanism of functional fine-tuning
Source: Nat Commun. 2019 Aug 15;10:3691. doi: 10.1038/s41467-019-11683-y (PMC6695400; doi:10.1038/s41467-019-11683-y)
Supplement: Supplementary file 1 — Supplementary Information [file 41467_2019_11683_MOESM1_ESM.pdf]

Diversity of oligomerization in *Drosophila* semaphorins suggests a mechanism of functional fine-tuning

Rozbesky *et al*.

Supplementary Figure 1

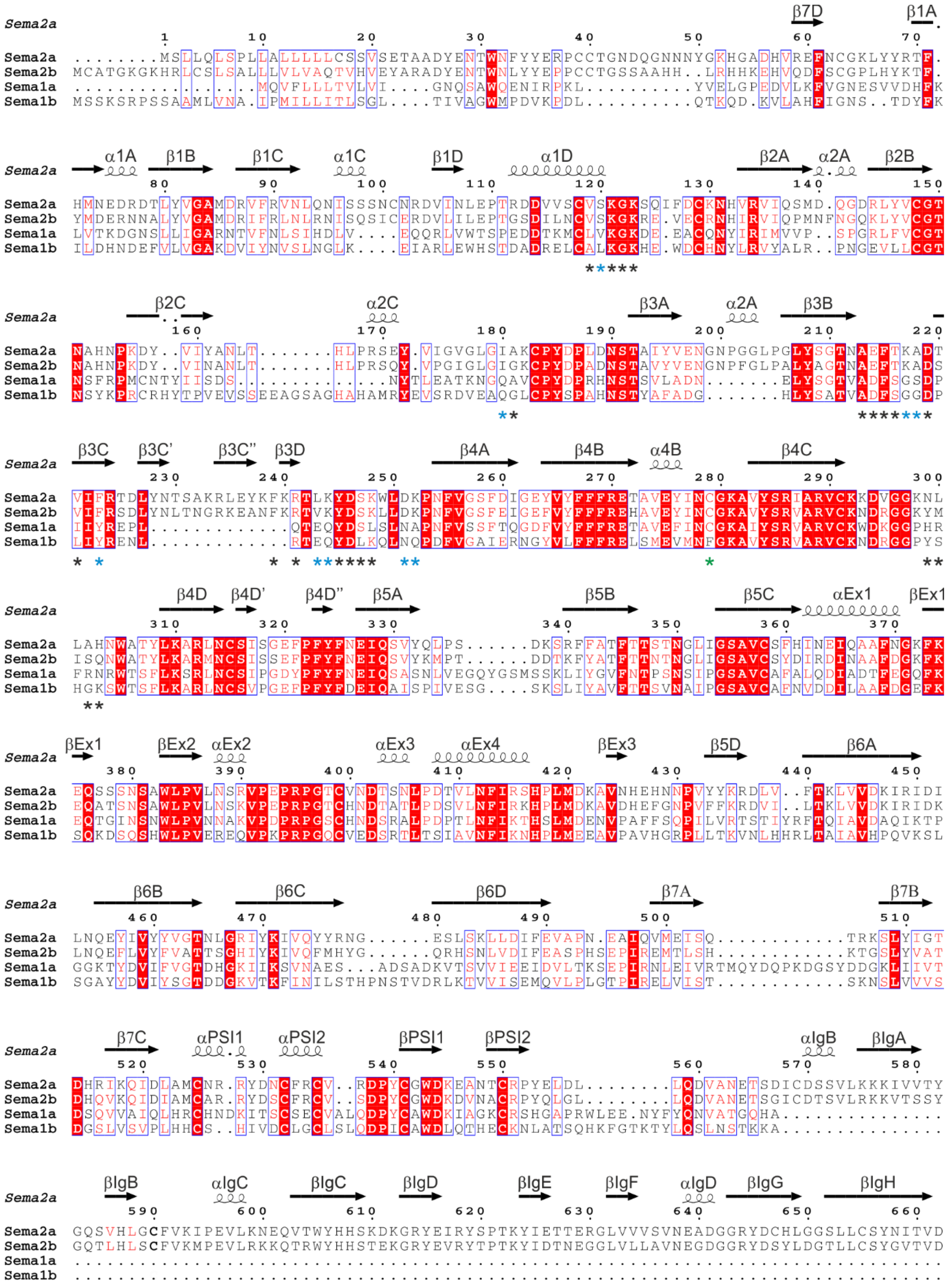

Supplementary Figure 1. Sequence alignment of *Drosophila* semaphorins

Structure-based sequence alignment of Sema1a$_{1-2}$, Sema1b$_{1-2}$, Sema2a$_{1-3}$ and Sema2b$_{1-3}$. Secondary structure elements and numbering for Sema2a are represented above the

sequences. The putative binding site for plexins is shown by blue and black asterisks. The residues were identified by superposition of *Drosophila* semaphorins with mouse Sema6A in complex with PlxnA2. The residues potentially driving specificity to the plexin receptor are indicated by blue asterisks. The position of cysteine involved in the interchain disulphide bond is indicated by green asterisk. The alignment was generated with Clustal Omega and formatted using ESPript.

Supplementary Figure 2

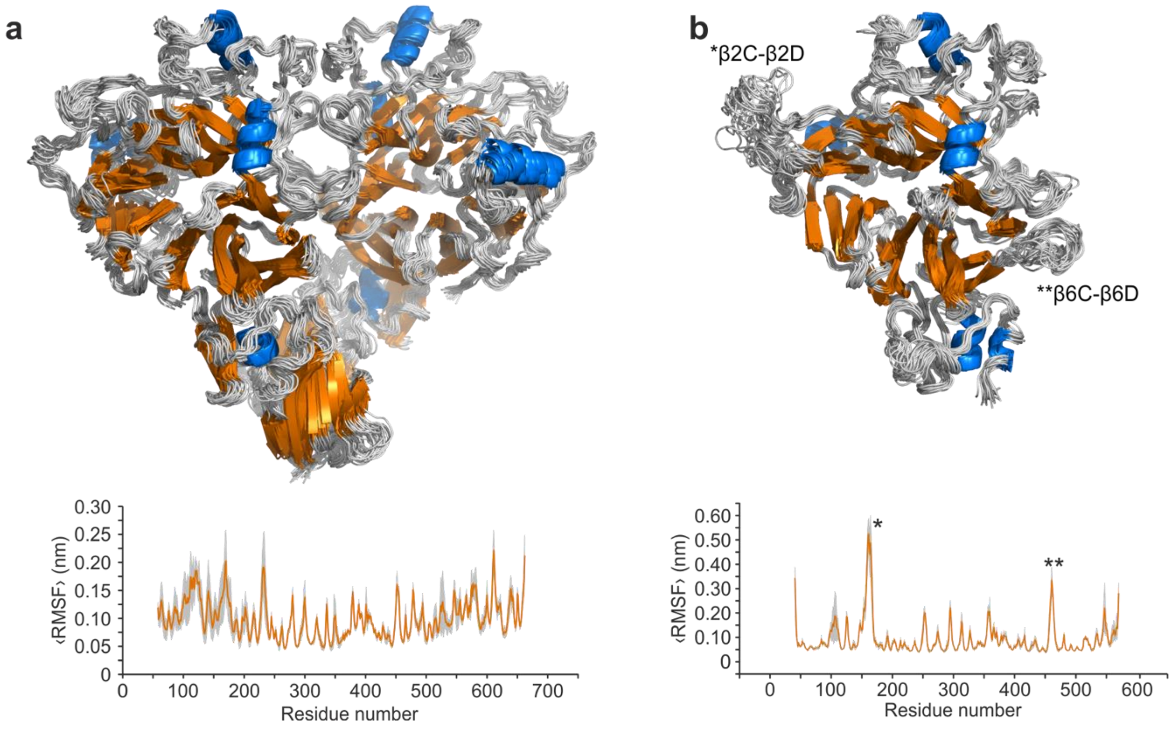

Supplementary Figure 2. Molecular dynamics simulation of dimeric Sema2a$_{1\text{-}3}$ and monomeric Sema1b$_{1\text{-}2}$

Superposition of 25 Cα-traced conformers extracted at 500 ps intervals in the molecular dynamics simulations revealed that Sema2a$_{1\text{-}3}$ (**a**) and Sema1b$_{1\text{-}2}$ (**b**) are relatively rigid proteins with low conformational flexibility. Average root mean square fluctuations (RMSF) of Cα atoms (bottom) showed two loops β2C-β2D and β6C-β6D (shown by asterisks) with a higher level of fluctuation relatively to the rest of protein. RMSF of Cα atoms (orange) was averaged from three independent simulations; the standard deviation is shown in grey. Source data are provided as a Source Data file.

Supplementary Figure 3

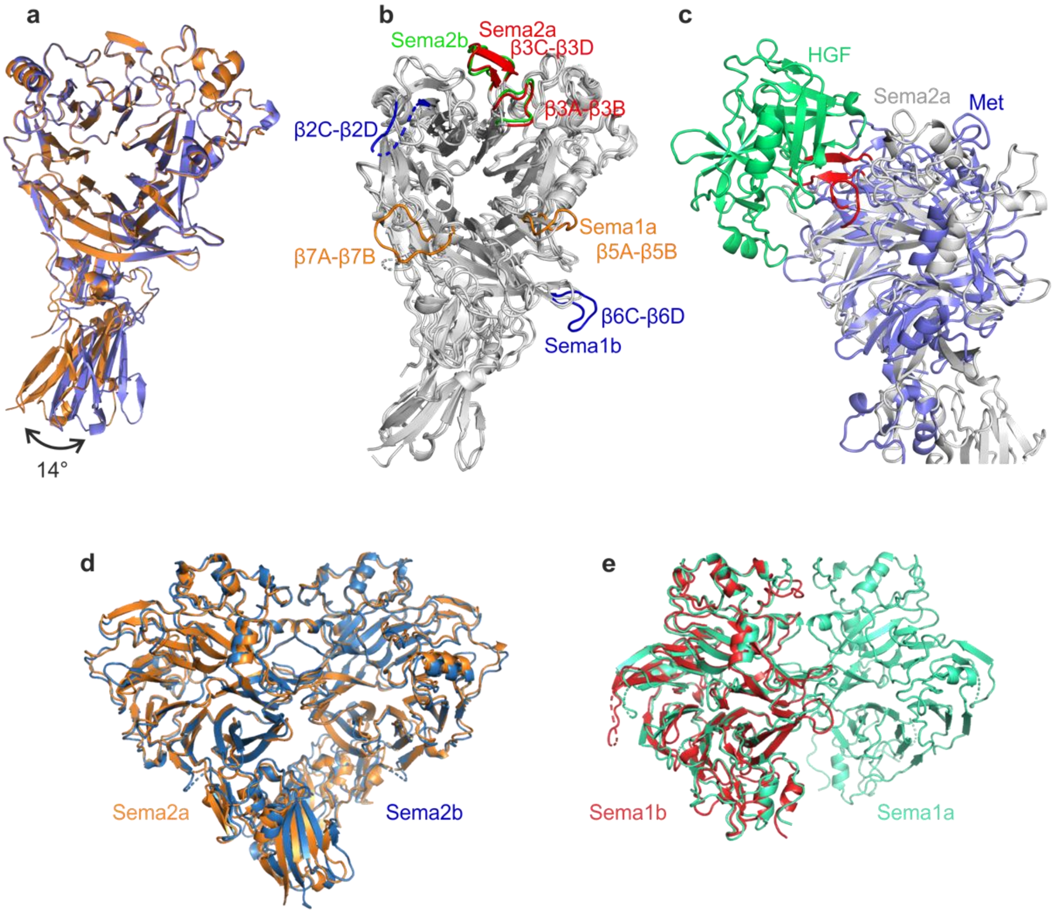

Supplementary Figure 3. Structural analysis of class1 and class 2 semaphorins
(**a**) Superposition of Sema2a$_{1-3}$ chain A (orange) and chain B (blue) via the sema domain shows the inter-domain flexibility between the sema and Ig-like domains. When considering only the sema domain, the superposition shows a difference of 14° about the junction between the PSI and Ig-like domains.
(**b**) Superposition of *Drosophila* semaphorin crystal structures reveals structural inter-class variations in the sema domain. In class 2 semaphorins, the β3A-β3B and β3C-β3D loops of blade 3 are uniquely extended from the bottom face of the β-propeller. In class 1 semaphorins, two loops β5A-β5B and β7A-β7B or β2C-β2D and β6C-β6D are also particularly extended from the bottom face of the sema domain in Sema1a or Sema1b, respectively. In addition, the β7A-β7B and β2C-β2D loops were not fully built in the crystal structures because of fragmentary electron density. Thus the β7A-β7B loop in Sema1a$_{1-2}$ in this figure was modelled by Modeller.
(**c**) Superposition of Sema2a$_{1-3}$ chain A (grey) and the MET receptor (purple) shows that the prominent and uniquely extended loops (β3A-β3B and β3C-β3D, shown in red) of Sema2a align with the major binding interface between the sema domain of the MET receptor and its ligand HGF-β (green) (pdb 1shy).
(**d-e**) Superposition of Sema2a$_{1-3}$ in orange and Sema2b$_{1-3}$ in blue (**d**) and Sema1a$_{1-2}$ in turquoise and Sema1b$_{1-2}$ in red (**e**).

Supplementary Figure 4

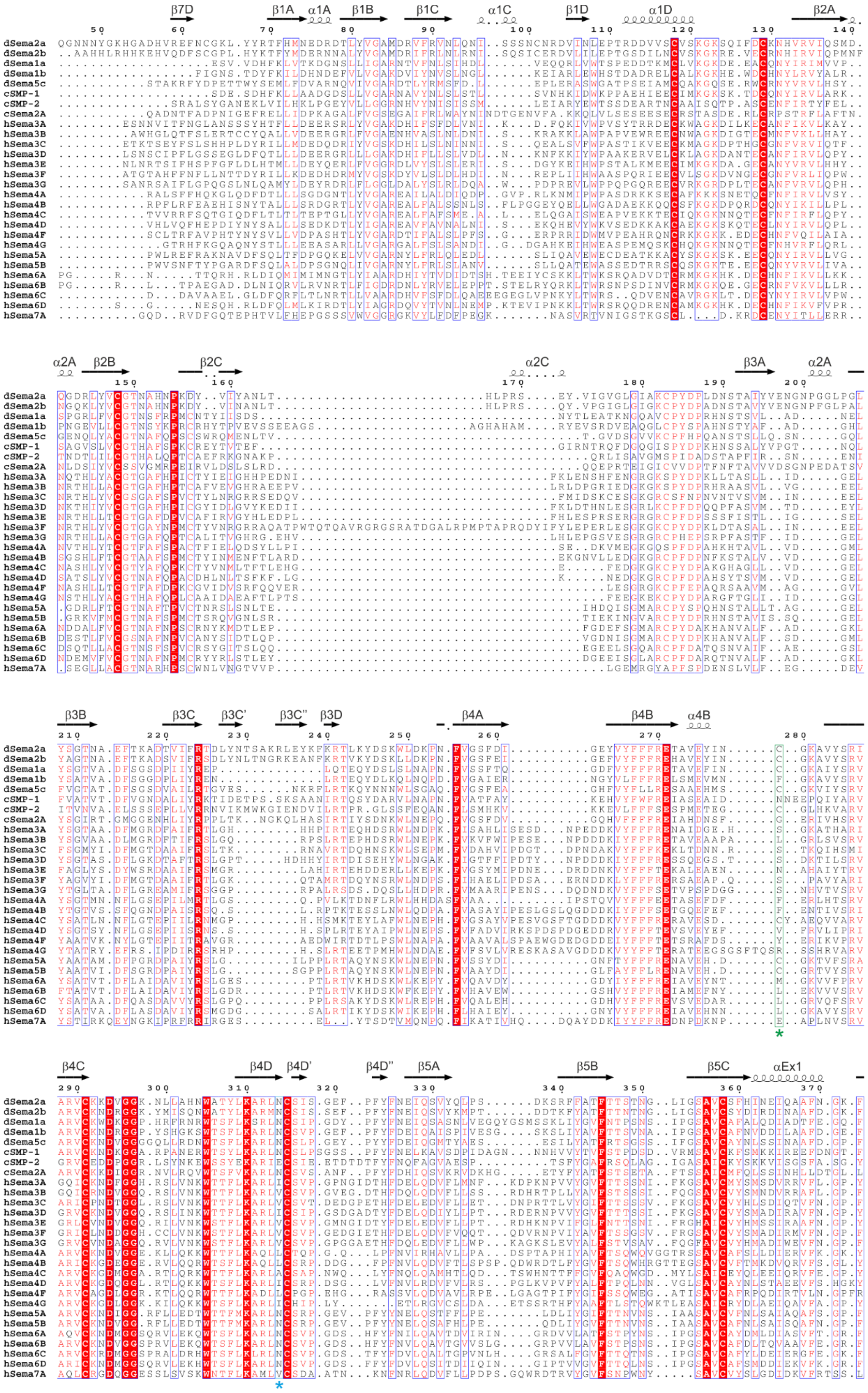

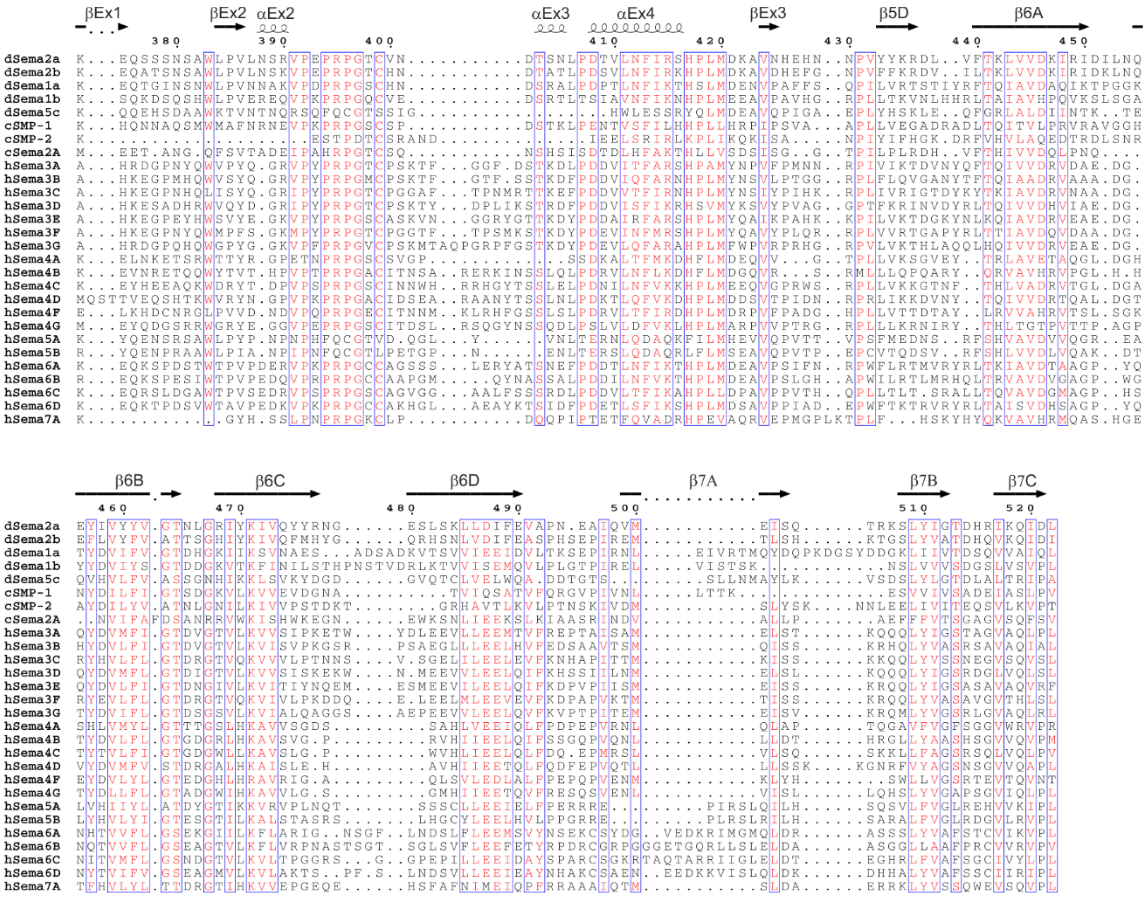

Supplementary Figure 4. Sequence alignment of *Drosophila*, *C. elegans* and human semaphorins

Structure-based sequence alignment of *Drosophila*, *C. elegans* and human semaphorins. Secondary structure elements and numbering for Sema2a are represented above the sequences. The position of cysteine involved in the interchain disulphide bond in Sema2a is indicated by green asterisk. The conserved glycosylation site with N-linked glycan involved in homodimerization is shown by blue asterisk. The alignment was generated with Clustal Omega and formatted using ESPript.

Supplementary Figure 5

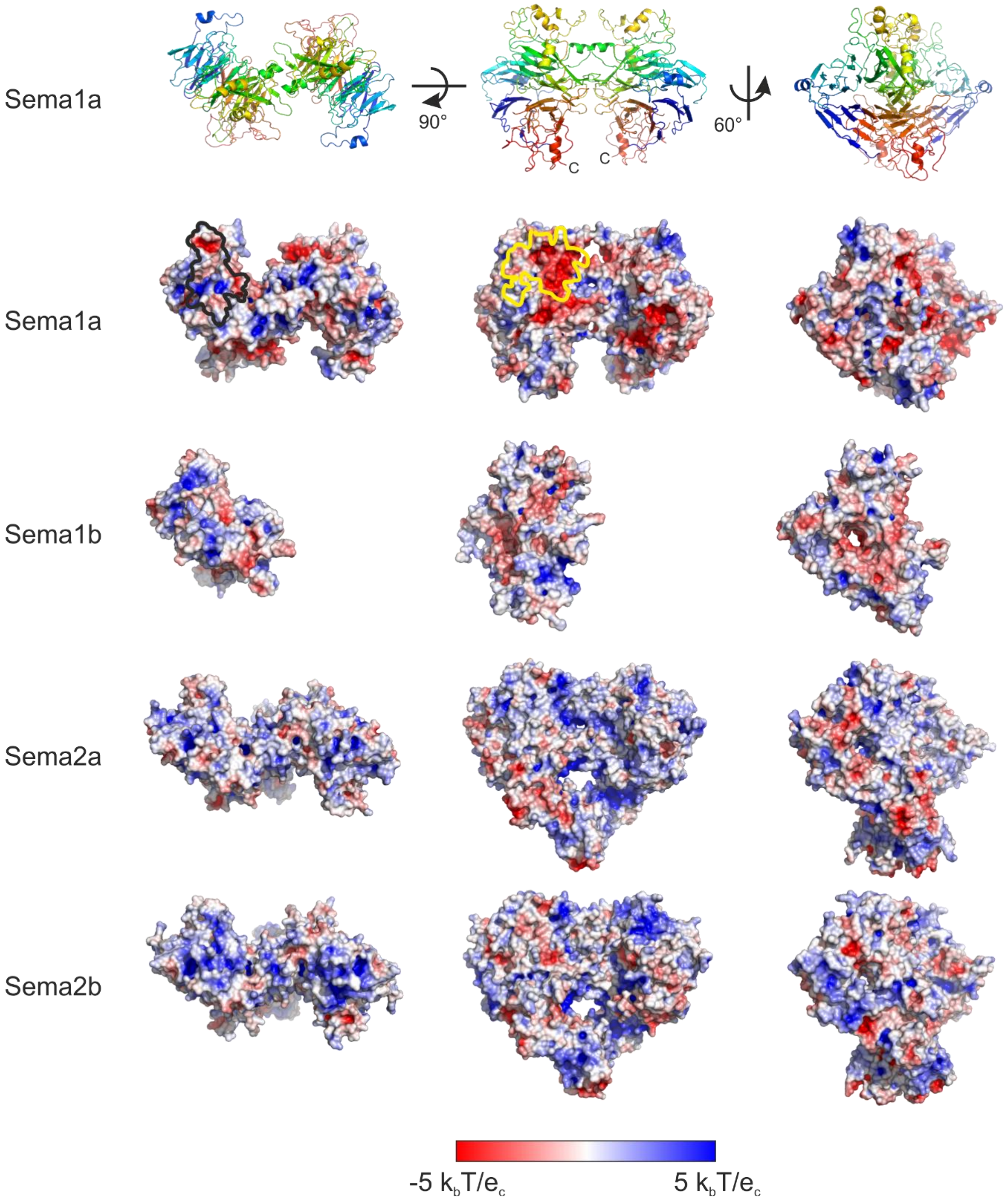

Supplementary Figure 5. Charge distribution on the surface of *Drosophila* semaphorins
Surface representation of *Drosophila* semaphorins coloured by electrostatic potential from -5k_bT/e_c (red) to +5k_bT/e_c (blue). The outline on the surface of Sema1a shows the putative plexin binding site (black) and the neuropilin binding site (yellow) as mapped from the crystal structures of the Sema6A-PlxnA2 and Sema3A-PlxnA2-Nrp1, respectively.

Supplementary Figure 6

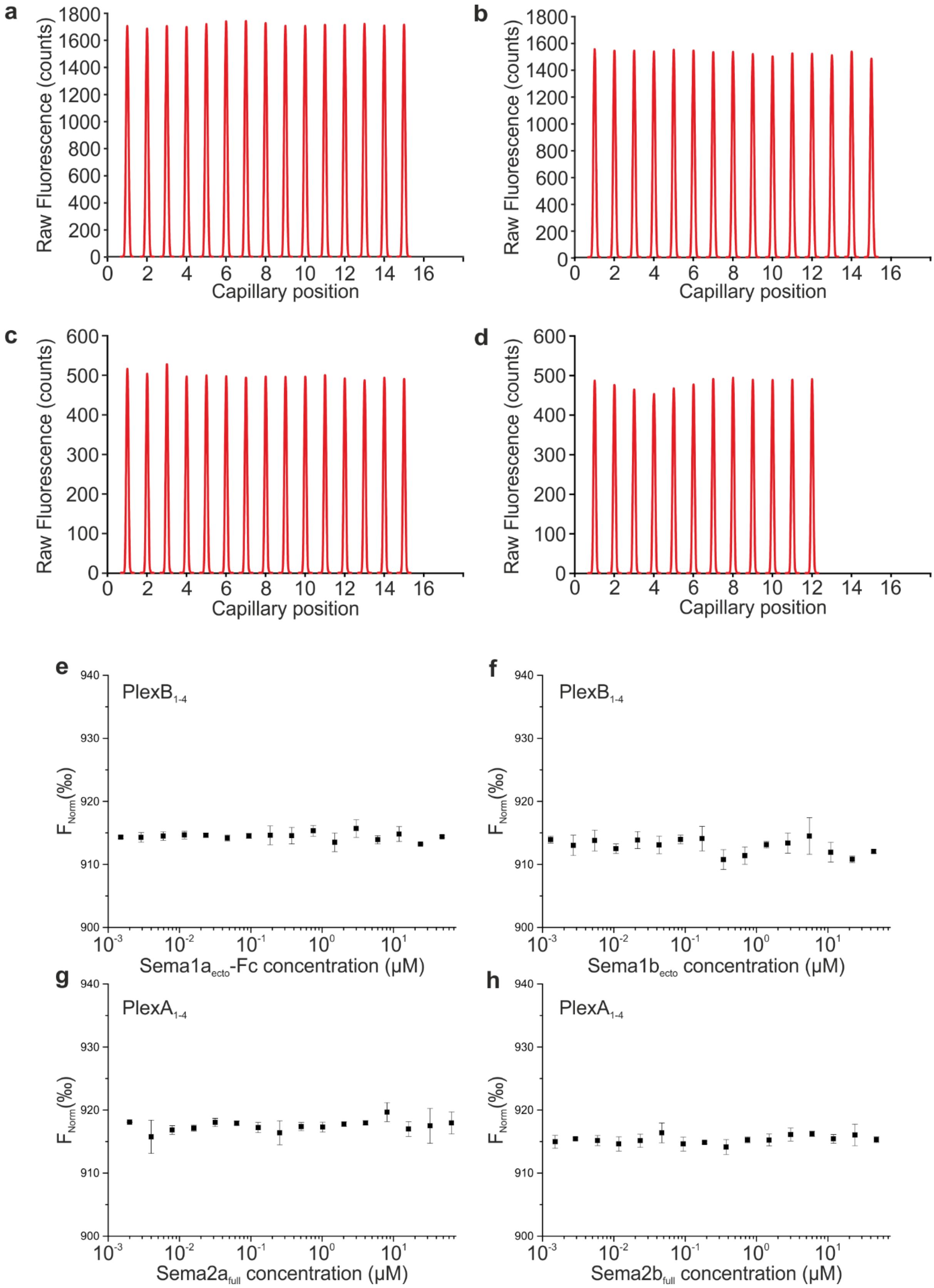

Supplementary Figure 6. Microscale thermophoresis binding experiment

(**a-d**) Representative capillary scans of PlexA$_{1\text{-}4}$-mVenus with Sema1a$_{ecto}$-Fc (**a**) and Sema1b$_{ecto}$ (**b**) or PlexB$_{1\text{-}4}$-mVenus with Sema2a$_{full}$ (**c**) and Sema2b$_{full}$ (**d**). The capillary scans show a symmetrical fluorescence peak demonstrating the good quality of the fluorescent samples, which show no non-specific binding to the surface of the capillaries. The fluorescence intensity was optimal for MST experiments (200-2500 fluorescent counts) and did not vary more than 10% between different capillaries.

(**e-h**) Fluorescence time traces recorded by the MST instrument show no binding between PlexB$_{1\text{-}4}$-mVenus and class 1 semaphorins (**e-f**) or PlexA$_{1\text{-}4}$-mVenus and class 2 semaphorins (**g-h**). Error bars represent s.d. of three technical replicates. Source data are provided as a Source Data file.

Supplementary Figure 7

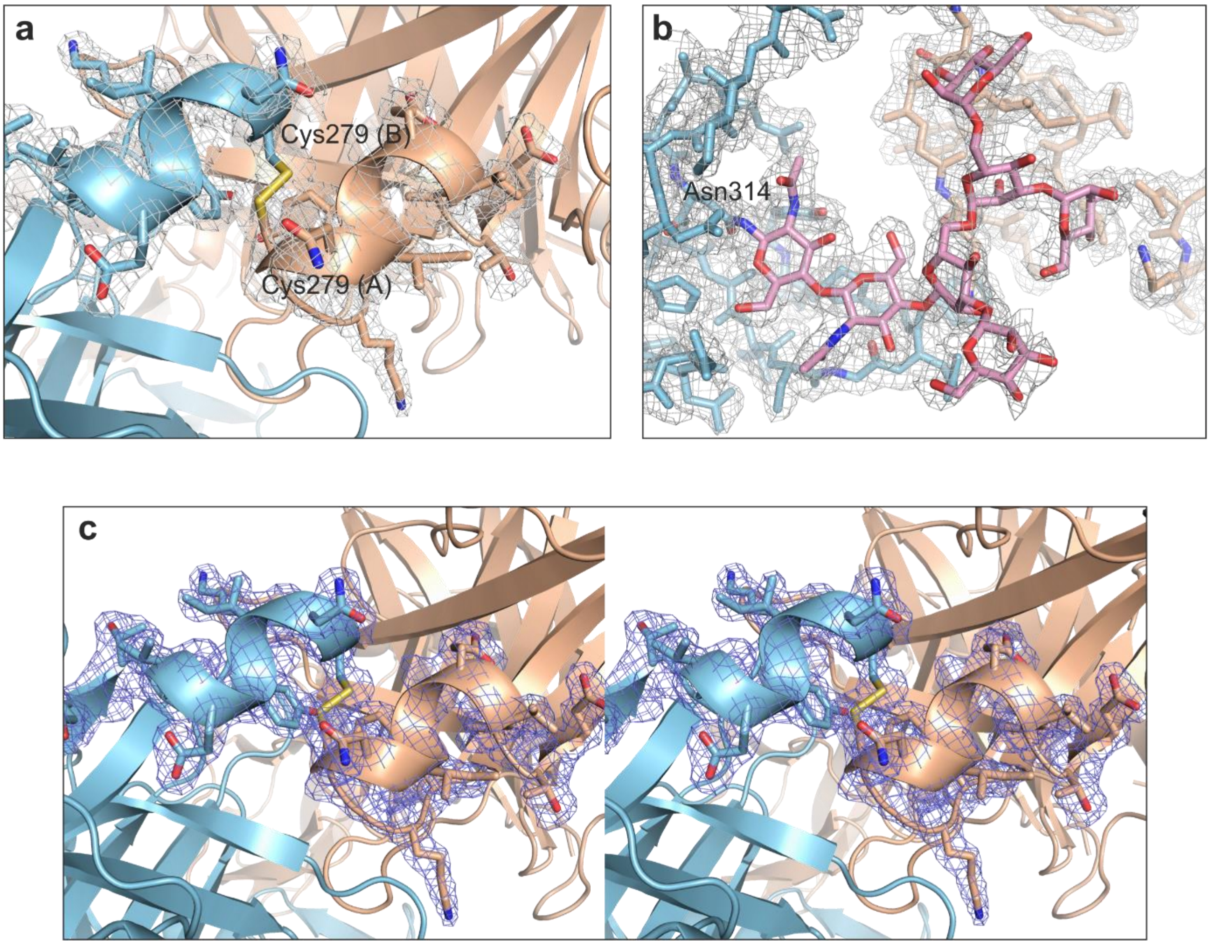

Supplementary Figure 7. Close-up views of the Sema2a$_{1\text{-}3}$ crystal structure showing the intermolecular disulphide bond and the N-linked glycans involved in the semaphorin homodimerization

(**a**) Close-up view of the intermolecular sema-to-sema disulphide bond formed by a cysteine 279 from each of the β4B-β4C loops of the opposing chains. The β4B-β4C loops are overlaid with the $2mF_o–DF_c$ map (calculated with Phenix for the final refined model) shown in grey at the contour level of 1.0 σ.

(**b**) Close-up view of the N-linked glycans at residue N314 in the Sema2a$_{1\text{-}3}$ crystal structure. The N-linked glycans of B chain (blue) form intermolecular interactions with residues of A chain (orange). The N-glycans are overlaid with the $2mF_o–DF_c$ map (calculated with Phenix for the final refined model) shown in grey at the contour level of 0.7 σ.

(**c**) Cross-eyed stereo image of (**a**).

Supplementary Figure 8

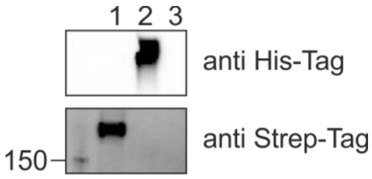

Supplementary Figure 8. *Drosophila* Sema1a and Sema2a do not form a heterodimer. HEK293T cells were transiently co-transfected with constructs encoding Sema2a$_{full}$-TwinStrep tag and Sema1a$_{ecto}$-His6 tag. Similarly to the purification of the Sema2a/2b heterodimer, we performed the same two-step affinity chromatography on the HisTrap column followed by TwinStrep tag purification on the StrepTactin column. Using Western blot analysis, we detected the homodimers in flow-through, Sema2a/2a (1) or Sema1a/1a (2), but not the Sema1a/2a heterodimer (3) suggesting that the heterodimerization between different semaphorin classes does not occur. Source data are provided as a Source Data file.

Supplementary Table 1 Data collection and refinement statistics

	**$Sema1a_{1-2}$ Anisotropy uncorrected**	**$Sema1a_{1-2}$ Anisotropy corrected**	**$Sema1b_{1-2}$ Anisotropy uncorrected**	**$Sema1b_{1-2}$ Anisotropy corrected**	**$Sema2a_{1-3}$**	**$Sema2b_{1-3}$**
**Data collection**						
Space group	$P\ 3_2$	$P\ 3_2$	$P\ 6_1\ 2\ 2$	$P\ 6_1\ 2\ 2$	$P\ 2_1\ 2_1\ 2_1$	$P\ 2_1\ 2_1\ 2_1$
Cell dimensions						
a, *b*, *c* (Å)	99.5, 99.5, 150.1	99.5, 99.5, 150.1	163.3, 163.3, 140.4	163.3, 163.3, 140.4	103.8, 110.0, 134.8	108.0, 145.6, 152.2
α, β, γ (°)	90, 90, 120	90, 90, 120	90, 90, 120	90, 90, 120	90, 90, 90	90, 90, 90
Resolution (Å)	49.74 - 3.60 (3.82 - 3.60)	49.74 - 3.60 (3.82 - 3.60)**	81.63 - 2.78 (2.90 - 2.78)	81.63 - 2.78 (2.90 - 2.78)**	57.46 - 1.96 (1.98 - 1.96)	65.67 - 2.33 (2.4 - 2.3)
R_{merge} (%)	23.3 (299.4)	19.2 (123.1)	11.7 (164.1)	11.1 (115.1)	17.5 (20.9)	18.2 (20.6)
$I / \sigma I$	5.00 (0.58)	6.2 (1.4)	10.93 (1.32)	12.60 (2.31)	11.51 (1.25)	10.45 (1.11)
Completeness (%)	96.98 (85.98)	79.46 (33.60)	96.74 (88.98)	79.79 (40.96)	99.60 (98.45)	98.42 (97.54)
Redundancy	6.1 (5.4)	6.0 (5.1)	12.6 (12.5)	11.7 (10.2)	14.8 (13.5)	7.6 (7.7)
CC1/2	0.992 (0.311)	0.992 (0.637)	0.998 (0.666)	0.998 (0.840)	0.998 (0.556)	0.996 (0.368)
**Refinement**						
No. reflections		15299		22592	110626	101313
R_{work} / R_{free} (%)		28.72/32.30		20.69/24.97	18.28/21.78	19.60/22.80
No. atoms		8000		4217	11373	11337
Protein		7916		4034	10004	9933
Ligand/ion		84		183	576	692
Water				793	712	
B-factors						
Protein		99.31		81.3	40.14	55.63
Ligand/ion		116.37		155.47	66.42	85.42
Water					43.87	49.40
R.m.s. deviations						
Bond lengths (Å)		0.003		0.003	0.004	0.004
Bond angles (°)		0.58		0.62	1.00	0.96

*Values in parentheses are for highest-resolution shell.

**Anisotropy correction of $Sema1a_{1-2}$ yielded an ellipsoidal resolution boundary with limits of 4.2, 4.2 and 3.2 Å along the a*, b*, and c* axes, respectively, while anisotropy correction of $Sema1b_{1-2}$ yielded an ellipsoidal resolution boundary with limits of 2.7, 2.7 and 3.5 Å along the a*, b*, and c* axes, respectively.

Supplementary Table 2 List of primers

Constructs	Primers
**Sema1a$_{ecto}$ (21-606)**	FORWARD: ATACCGGTAATATACGACCAAAAC
	REVERSE: ATCGTACGCTCCACAGTGTACTGGGC
**Sema1a$_{1-2}$ (21-602)**	FORWARD: ATACCGGTAATATACGACCAAAAC
	REVERSE: ATCGTACGCTGGGCGTTGAT
**Sema1b$_{ecto}$ (37-659)**	FORWARD: ATACCGGTGACGTGAAGCCTG
	REVERSE: ATGGTACCGCTGGGCGTTG
**Sema1b$_{1-2}$ (37-602)**	FORWARD: ATACCGGTGACGTGAAGCCTG
	REVERSE: ATGGTACCCGAGCCCACATTG
**Sema2a$_{full}$ (26-724)**	FORWARD: AAACCGGTGACTACGAGAACACC
	REVERSE: TTGGTACCGACATTGGGCTTACG
**Sema2a$_{1-3}$ (27-671)**	FORWARD: CTCACCGGTTACGAGAACACCTGGAACTTC
	REVERSE: CGGGGTACCCTTGTTCGGCGGAGTGCATC
**Sema2b$_{full}$ (34-736)**	FORWARD: AAACCGGTGATTATGAGAACACC
	REVERSE: TTCGTACGGACCAAAGCATCGTTG
**Sema2b$_{1-3}$ (33-679)**	FORWARD: ATACCGGTGAGAACACCTGGAATCTA
	REVERSE: ATCGTACGCTTCTGCGATGGCGGCGA
**PlexA$_{1-4}$ (28-730)**	FORWARD: ATCCCGGGCAAACCCTGGC
	REVERSE: ATGGTACCCGTTGGACAAAATCC
**PlexB$_{1-4}$ (35-730)**	FORWARD: ATACCGGTGAAGAACTGCCG
	REVERSE: AAGGTACCGGGGCAGTGACCAACAG
**Sema3A (26-730)**	FORWARD: GGGGACCGGTAAGAACAATGTGCCAAGA
	REVERSE: GGAAGGTACCGTCCCTTTTCCACACTTGTTCAC
**Sema3C (22-711)**	FORWARD: GGGGACCGGTTCTTCCCAGCCCCAAGCAA
	REVERSE: GAGAGGTACCGTCTTTGCAGTACTGATTGATGAG
**Sema1a C227S**	FORWARD: CCGCCGTTGAGTTTATCAACAGTGGCAAGGCGATTTATTCGCGC
	REVERSE: GCGCGAATAAATCGCCTTGCCACTGTTGATAAACTCAACGGCGG
**Sema1b F254C**	FORWARD: GAAGTCATGAACTGTGGCAAGGCC
	REVERSE: GGCCTTGCCACAGTTCATGACTTC
**Sema2a C279S**	FORWARD: GAAACCGCCGTGGAATACATCAACTCCGGCAAGGCTGTCTATTCGCGCATCGC
	REVERSE: GCGATGCGCGAATAGACAGCCTTGCCGGAGTTGATGTATTCCACGGCGGTTTC
**Sema2b C334S**	FORWARD: CGAACATGCCGTTGAGTACATAAATTCCGGAAAAGCCGTCTACTCCCGAG
	REVERSE: CTCGGGAGTAGACGGCTTTTCCGGAATTTATGTACTCAACGGCATGTTCG
