# Supplementary material for: Diversity of oligomerization in Drosophila semaphorins suggests a mechanism of functional fine-tuning
Source: Nat Commun. 2019 Aug 15;10:3691. doi: 10.1038/s41467-019-11683-y (PMC6695400; doi:10.1038/s41467-019-11683-y)
Supplement: Supplementary file 3 — Reporting Summary [file 41467_2019_11683_MOESM3_ESM.pdf]

nature research

Corresponding author(s): Daniel Rozbesky, Yvonne Jones

Last updated by author(s): Jul 22, 2019

Reporting Summary

Nature Research wishes to improve the reproducibility of the work that we publish. This form provides structure for consistency and transparency in reporting. For further information on Nature Research policies, see Authors & Referees and the Editorial Policy Checklist.

Statistics

For all statistical analyses, confirm that the following items are present in the figure legend, table legend, main text, or Methods section.

n/a	Confirmed	
☐	☒	The exact sample size (*n*) for each experimental group/condition, given as a discrete number and unit of measurement
☐	☒	A statement on whether measurements were taken from distinct samples or whether the same sample was measured repeatedly
☒	☐	The statistical test(s) used AND whether they are one- or two-sided<br>*Only common tests should be described solely by name; describe more complex techniques in the Methods section.*
☒	☐	A description of all covariates tested
☒	☐	A description of any assumptions or corrections, such as tests of normality and adjustment for multiple comparisons
☐	☒	A full description of the statistical parameters including central tendency (e.g. means) or other basic estimates (e.g. regression coefficient) AND variation (e.g. standard deviation) or associated estimates of uncertainty (e.g. confidence intervals)
☒	☐	For null hypothesis testing, the test statistic (e.g. *F*, *t*, *r*) with confidence intervals, effect sizes, degrees of freedom and *P* value noted<br>*Give P values as exact values whenever suitable.*
☒	☐	For Bayesian analysis, information on the choice of priors and Markov chain Monte Carlo settings
☒	☐	For hierarchical and complex designs, identification of the appropriate level for tests and full reporting of outcomes
☒	☐	Estimates of effect sizes (e.g. Cohen's *d*, Pearson's *r*), indicating how they were calculated

Our web collection on statistics for biologists contains articles on many of the points above.

Software and code

Policy information about availability of computer code

Data collection	GDA (GPLv3) - synchrotron data collection (Diamond Light Source)<br>ASTRA Software (Wyatt Technology) - SEC MALS data acquisition<br>Monolith Data Acquisition software for NT.115 (Nanotemper) - Microscale thermophoresis data acquisition<br>Bio-Rad Image Lab Software - SDS PAGE gels and Western blot imaging

Data analysis	XIA2 (v.0.5.771) - automated reduction of X-ray diffraction data<br>PHENIX (v1.15rc3-3435) - analysis, validation and manipulation of X-ray diffraction data<br>CCP4 (v.7.0.071) - - analysis, validation and manipulation of X-ray diffraction data<br>COOT (v.0.8.9.1) - building and validation of atomic models<br>STARANISO SERVER - diffraction anisotropy correction (http://staraniso.globalphasing.org/cgi-bin/staraniso.cgi)<br>HKL2000 (v708c) - x-ray diffraction data processing<br>CLUSTAL OMEGA - multiple sequence alignment program (https://www.ebi.ac.uk/Tools/msa/clustalo/)<br>PDBEFOLD - pairwise comparison and 3D alignment (http://www.ebi.ac.uk/msd-srv/ssm/)<br>PDBePISA - analysis of macromolecular interfaces (http://www.ebi.ac.uk/pdbe/pisa/)<br>APBS (v2.1)- electrostatics calculation<br>SHP - structure-based phylogenetic tree calculation<br>PYMOL (Schrodinger, LLC, v1.8.6.2) - molecular visualization system<br>ESPRIPT 3.0 - sequence alignment<br>COREL DRAW X6 (Corel Corporation) - vector graphics editor<br>ASTRA Software (Wyatt Technology - v6.1.2) - SEC MALS Data analysis<br>MO AFFINITY ANALYSIS (Nanotemper - v2.1.3) - Microscale thermophoresis data analysis<br>GROMACS (v5.1.2)- molecular dynamics simulation<br>Modeller (v9.19) - homology modelling of protein 3D structures<br>OriginPro (v9.1) - plotting the data from molecular dynamics simulation

For manuscripts utilizing custom algorithms or software that are central to the research but not yet described in published literature, software must be made available to editors/reviewers. We strongly encourage code deposition in a community repository (e.g. GitHub). See the Nature Research guidelines for submitting code & software for further information.

Data

Policy information about availability of data

All manuscripts must include a data availability statement. This statement should provide the following information, where applicable:

- Accession codes, unique identifiers, or web links for publicly available datasets
- A list of figures that have associated raw data
- A description of any restrictions on data availability

Structure factors and coordinates have been deposited in the Protein Data Bank with identification numbers PDB: 6QP9, 6FKK, 6QP7 and 6QP8. Source data underlying Fig2, Fig5B, Fig5C, Fig5F, Fig5G, Supplementary Fig2 and Supplementary Fig8 are available as a Source Data file. Other data and materials are available upon request from the corresponding authors.

Field-specific reporting

Please select the one below that is the best fit for your research. If you are not sure, read the appropriate sections before making your selection.

☒ Life sciences ☐ Behavioural & social sciences ☐ Ecological, evolutionary & environmental sciences

For a reference copy of the document with all sections, see nature.com/documents/nr-reporting-summary-flat.pdf

Life sciences study design

All studies must disclose on these points even when the disclosure is negative.

Sample size	The variability of the effect size induced by the ligand was determined by performing three individual replicates.
Data exclusions	Data points were excluded when there was a technical mistake in the ligand preparation or during the procedure of the experiment
Replication	All binding experiments were performed in triplicates including preparation of the dilutions. All attempts at replication were successful.
Randomization	Randomization was not relevant to our study.
Blinding	Blinding was not relevant to our study.

Reporting for specific materials, systems and methods

We require information from authors about some types of materials, experimental systems and methods used in many studies. Here, indicate whether each material, system or method listed is relevant to your study. If you are not sure if a list item applies to your research, read the appropriate section before selecting a response.

Materials & experimental systems

n/a	Involved in the study
	☒ Antibodies
	☒ Eukaryotic cell lines
☒	Palaeontology
☒	Animals and other organisms
☒	Human research participants
☒	Clinical data

Methods

n/a	Involved in the study
☒	ChIP-seq
☒	Flow cytometry
☒	MRI-based neuroimaging

Antibodies

Antibodies used

Precision Protein™ StrepTactin-HRP Conjugate (Biorad, cat. no. 1610380)
Penta-His Antibody, BSA free (Qiagen. cat. no. 34660, lot. no. 160017634)
Anti-mouse IgG (Fc specific) - Peroxidase polyclonal goat antibody (Sigma, cat. no. A0168, lot. no. 068M4764V)

Validation

Each primary antibody was validated for the specific detection of the relevant epitope tag.

Eukaryotic cell lines

Policy information about cell lines

Cell line source(s)

HEK293T (ATCC CRL-3216)
HEK293S-GnTI (ATCC CRL-3022)
High Five cells (Trichoplusia ni) (ATCC CRL-10859)

Authentication

None of these cell lines were explicitly authenticated.

Mycoplasma contamination

All cell lines tested negative for mycoplasma.

Commonly misidentified lines
(See ICLAC register)

No ILAC listed cell lines that are commonly misidentified were used in this study.
